# *Melav2*, an *elav*-like gene, is essential for spermatid differentiation in the flatworm *Macrostomum lignano*

**DOI:** 10.1186/1471-213X-9-62

**Published:** 2009-12-08

**Authors:** Kiyono Sekii, Willi Salvenmoser, Katrien De Mulder, Lukas Scharer, Peter Ladurner

**Affiliations:** 1 Department of Evolutionary Biology, Zoological Institute, University of Basel, Basel, Switzerland; 2 Department of Ultrastructural Research and Evolutionary Biology, Institute of Zoology, University of Innsbruck, Innsbruck, Austria; 3 Department of Biology, University of Ghent, Ghent, Belgium

## Abstract

**Background:**

Failure of sperm differentiation is one of the major causes of male sterility. During spermiogenesis, spermatids undergo a complex metamorphosis, including chromatin condensation and cell elongation. Although the resulting sperm morphology and property can vary depending on the species, these processes are fundamental in many organisms. Studying genes involved in such processes can thus provide important information for a better understanding of spermatogenesis, which might be universally applied to many other organisms.

**Results:**

In a screen for genes that have gonad-specific expression we isolated an *elav*-like gene, *melav2*, from *Macrostomum lignano*, containing the three RNA recognition motifs characteristic of *elav*-like genes. We found that *melav2 *mRNA was expressed exclusively in the testis, as opposed to the known *elav *genes, which are expressed in the nervous system. The RNAi phenotype of *melav2 *was characterized by an aberrant spermatid morphology, where sperm elongation often failed, and an empty seminal vesicle. *Melav2 *RNAi treated worms were thus male-sterile. Further analysis revealed that in *melav2 *RNAi treated worms precocious chromatin condensation occurred during spermatid differentiation, resulting in an abnormally tightly condensed chromatin and large vacuoles in round spermatids. In addition, immunostaining using an early-spermatid specific antibody revealed that *melav2 *RNAi treated worms had a larger amount of signal positive cells, suggesting that many cells failed the transition from early spermatid stage.

**Conclusion:**

We characterize a new function for *elav*-like genes, showing that *melav2 *plays a crucial role during spermatid differentiation, especially in the regulation of chromatin condensation and/or cell elongation.

## Background

Failure of spermatogenesis is one of the major causes of male sterility. Many cases of human infertility are associated with low sperm production (oligozoospermia), poor sperm motility (asthenozoospermia) and abnormal sperm morphology (teratozoospermia) [1]. Also, it has been shown that abnormalities of sperm chromatin, which is important for properly transmitting genetic information to offspring, can often be observed in cases of infertility [2]. Although spermatid cells undergo a complex metamorphosis in a species-specific manner, resulting in various types of sperm morphology and other traits depending on species, fundamental processes such as the reorganization of the nucleus, cell organelles, and cell shape are found in most organisms [3]. Thus studying genes involved in such processes can provide important information for a better understanding of spermatogenesis, which may also be applied to many other organisms.

*Macrostomum lignano *(Macrostomorpha, Rhabditophora, Platyhelminthes) is a simultaneously hermaphroditic flatworm, namely with male and female gonads within one individual, and a suitable model organism for gametogenesis research. An EST database is available as a source for gene information http://flatworm.uibk.ac.at/macest/[4]. In addition, basic experimental techniques for gene analysis such as *in situ *hybridization and gene specific RNA interference (RNAi) are already established [5,6]. Monoclonal antibodies against various types of cells are also available for detailed analysis of tissues [7]. The biggest advantage of *M. lignano *for spermatogenesis research is its transparency, which allows non-invasive observation of cells and tissues in live animals using light microscopy and thus allows very efficient screening of RNAi phenotypes. Finally, their frequent copulation and short generation time make it easy to examine mating behavior and the degree of reproductive contribution to the next generation [8]. Also a lot of research has been done in terms of evolutionary biology, such as sex allocation adjustment, sexual conflict, and sperm competition [9-12]. Thus studying *M. lignano *allows the comprehensive understanding of spermatogenesis, not only at the developmental level but also its significance in evolutionary aspects.

In *M. lignano*, the testes mainly consist of male germ cells such as spermatogonia, spermatocytes, spermatids, and sperm. A thin layer of tunica cells encloses the testes. After meiosis, spermatids remain in four-cell clusters with cytoplasmic connections until just before the completion of sperm maturation [13]. Mature sperm have a complex morphology, with distinct parts easily observed in the microscope. From anterior to posterior, sperm have a feeler, a body which ends in a pair of bristles, and a shaft which ends in a brush [14]. Within the shaft the nucleus is condensed into connected packages of compact chromatin. The overall appearance of the sperm nucleus can be compared to connected railway carriages [13]. This morphology will be referred to as "train-shape" in this manuscript. Sperm differentiation starts with the development of the anterior part which is followed by the elongation of spermatids and then the nucleus becomes enclosed in the shaft of the mature sperm. Here we reserve the term 'sperm' for cells that have completed spermatogenesis and call the aberrant cells of the RNAi phenotype 'aberrant spermatids'.

In the process of spermiogenesis, post-transcriptional control of mRNA is very important [15,16]. In an elongating spermatid, the nucleus has to be condensed into a compact shape, which causes cessation of transcription. Therefore, transcription of genes that are necessary for later stages has to be completed before chromatin condensation, but their translation needs to be controlled until they are needed. For this post-transcriptional control, such as transport, translational repression and storage of mRNAs, the involvement of various types of RNA binding proteins has been reported [15-19]. For example, during spermiogenesis DNA-binding histones are gradually replaced by transition proteins (Tnp1 and Tnp2) and then protamines (Prm1 and Prm2), and it is suggested that protamine-1 mRNA binding protein (PRBP) has a role for proper translational activation of *prm-1 *mRNA [17].

*Elav *genes are RNA binding proteins that are characterized by three RNA recognition motifs (RRMs) and a hinge region between the second and the third RRM [20,21]. RRMs are the most common protein domains found in all kingdoms and each RRM consists of 80-90 amino acids containing two conserved sequences called RNP-1 and RNP-2 [22-25]. Structurally, a RRM has two α helices and four anti-parallel β strands, forming two β-α-β structures. It is suggested that proteins containing one or several RRMs are capable of interacting with RNA molecules [23-25]. Molecular functions of the *elav *gene family are quite diverse, including mRNA stability, splicing, translatability and transport [20,26].

In this paper, we study a *Macrostomum elav*-like gene, *melav2 *(*Macrostomum elav*-like gene **2**), using differential interference contrast microscopy, *in situ *hybridization, monoclonal antibodies, histology, and electron microscopy. We found that *melav2 *is expressed exclusively in the testes. Moreover, we show that *melav2 *RNA interference causes aberrant spermatid morphology, abnormal chromatin condensation, and an empty seminal vesicle leading to male-sterility. We thus prove that *melav2 *plays a crucial role for proper sperm differentiation and male fertility in *M. lignano*.

## Results

### *Melav2 *is an *elav*-like gene encoding three RNA recognition motifs

*Melav2 *was identified and isolated during the process of screening for gonad-specific genes in *M. lignano*. We found this gene in our EST database with the keyword '*sex-lethal *(*sxl*)'. *Sxl *is a well-known gene involved in sex determination in *Drosophila melanogaster*, although its function is not conserved among all insects [27-31]. However, we found that, as described in detail below, *melav2 *had a higher similarity with *elav *genes than with *sxl*. We therefore named it *melav2 *(***M****acrostomum elav*-like gene **2**) to distinguish it from *melav1*, another *elav*-like gene present in our EST database. Given the scope of our paper we focused on *melav2*. The entire open reading frame of this gene was obtained from the EST clone ANGU919. This gene was predicted to encode 422 amino acids.

**Figure 1:**
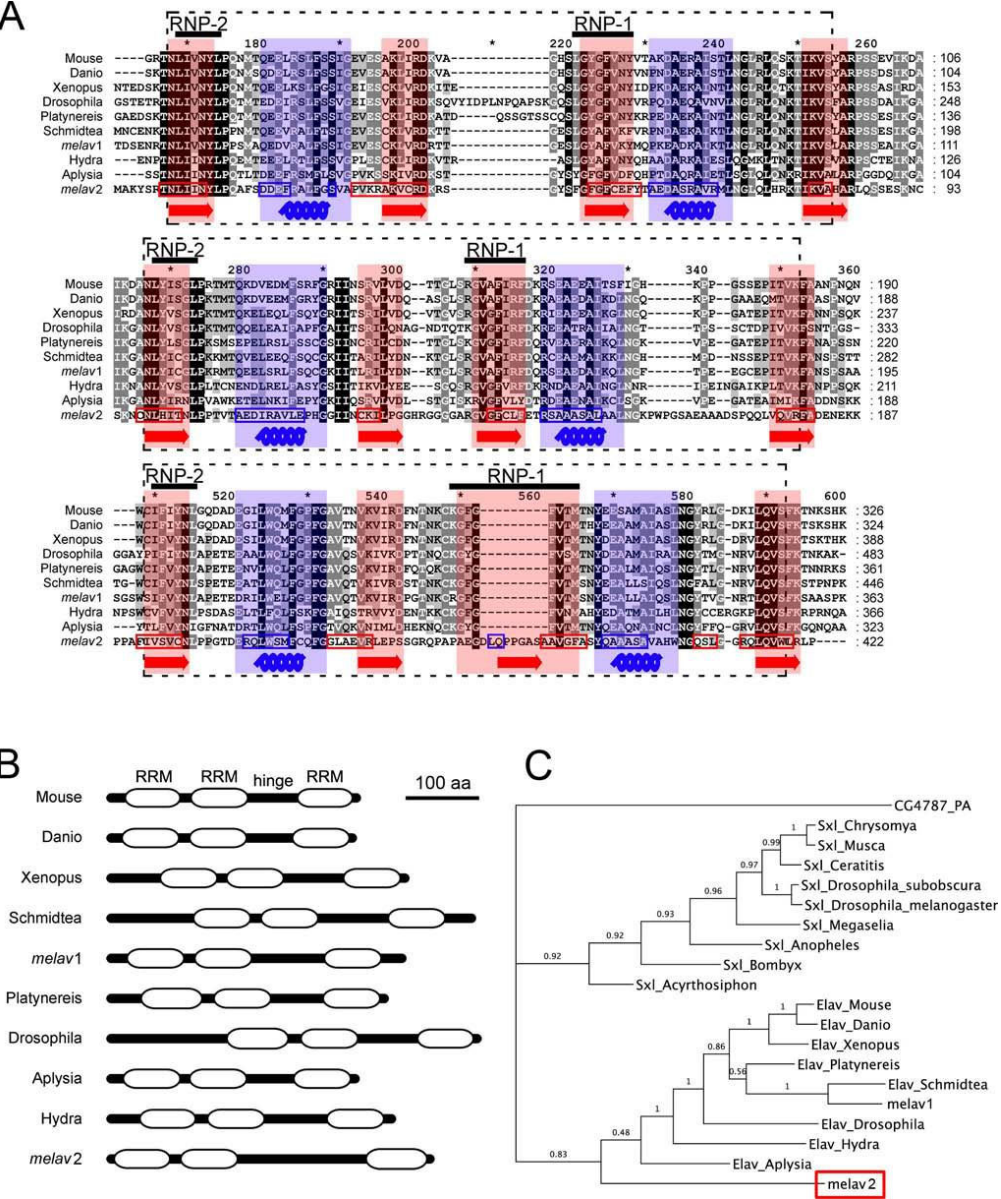
**Comparison of *melav2 *and other *elav *family genes**. (A) Alignments of the *melav2 *amino acid sequence with other *elav*-like genes from different organisms. Three RNA recognition motifs (RRM) are present in all *elav*-like genes (each indicated by a dashed line box). Each RRM has two conserved sequences RNP-1 and RNP-2. Structurally, RRM consists of two alpha helixes (highlighted in blue and blue coil) and four beta sheets (highlighted in red and red arrow). The predicted corresponding sequence of *melav2 *is shown in blue and red boxes, respectively. (B) Predicted domain structure by the SMART program. Each box indicates an RRM. *Melav2 *had a similar gene structure that is similar to other *elav *family genes, namely three RRMs with a hinge region between the second and the third RRM. (C) Phylogenetic tree of *melav2*, other *elav *genes, and *sxl *genes from different organisms. *Melav2 *was categorized into the *elav *gene family. Numbers show the Bayesian posterior probability (with values > 0.95 representing good nodal support). For accession numbers see materials and methods. Mouse: *Mus musculus*, Xenopus: *Xenopus laevis*, Danio: *Danio rerio*, Drosophila: *Drosophila melanogaster*, Aplysia: *Aplysia californica*, Platynereis: *Platynereis dumerilii*, Schmidtea: *Schmidtea mediterranea*, Hydra: *Hydra magnipapillata*, *melav1*: another *elav*-like gene from *M. lignano *(see the discussion), Musca: *Musca domestica*, Ceratitis: *Ceratitis capitata*, Chrysomya: *Chrysomya rufifacies*, Megaselia: *Megaselia scalaris*, Anopheles: *Anopheles gambiae*, Acyrthosiphon: *Acyrthosiphon pisum*, Bombyx: *Bombyx mori*, CG4787-PA: a protein from *D. melanogaster *as an outgroup.

Using BLASTX analysis, we found that *melav2 *has a high similarity with the Elav/Hu gene family, which is characterized by three RRMs with a hinge region between the second and the third RRM. Multiple alignments with other *elav*-like genes (Figure 1A) revealed that the first and second RRM of *melav2 *are relatively conserved whereas the third RRM does not show such a high homology.

The structure of the RRM is important for the interaction with RNA. We therefore examined the structural similarity of *melav2 *and other *elav *genes. An RRM contains two α helices and four anti-parallel β strands, forming two β-α-β structures [22-25]. Structural analysis using the SWISS-MODEL program revealed that *melav2 *has two such β-α-β structures in each RRM region and that they correspond to the β-α-β region of other *elav *genes (Figure 1A). In the third RRM of *melav2*, however, the β-α-β pattern was less distinct. We further analyzed *melav2 *using the SMART program for identification and annotation of protein domains (Figure 1B). According to this program, *melav2 *was considered to have three RRMs, corroborating the similar overall gene structure to other *elav *genes. The e-value of the first and second RRM was 4.89e-18 and 2.16e-10, respectively, and the e-value of the third RRM was 4.61e-05. All these e-values were for the RRM in the SMART database (SMART accession number SM00360).

BLASTX analysis also showed a certain similarity between *melav2 *and another *elav *related gene, *sxl *(data not shown). Structurally, *sxl *has two RRMs. The third RRM of *melav2 *was not well conserved compared to other *elav*-like genes, but despite the similarity with *sxl*, the phylogenetic analysis revealed that *melav2 *seemed to belong to the *elav*-like gene family (Figure 1C).

### *Melav2 *is expressed exclusively in the testes

**Figure 2:**
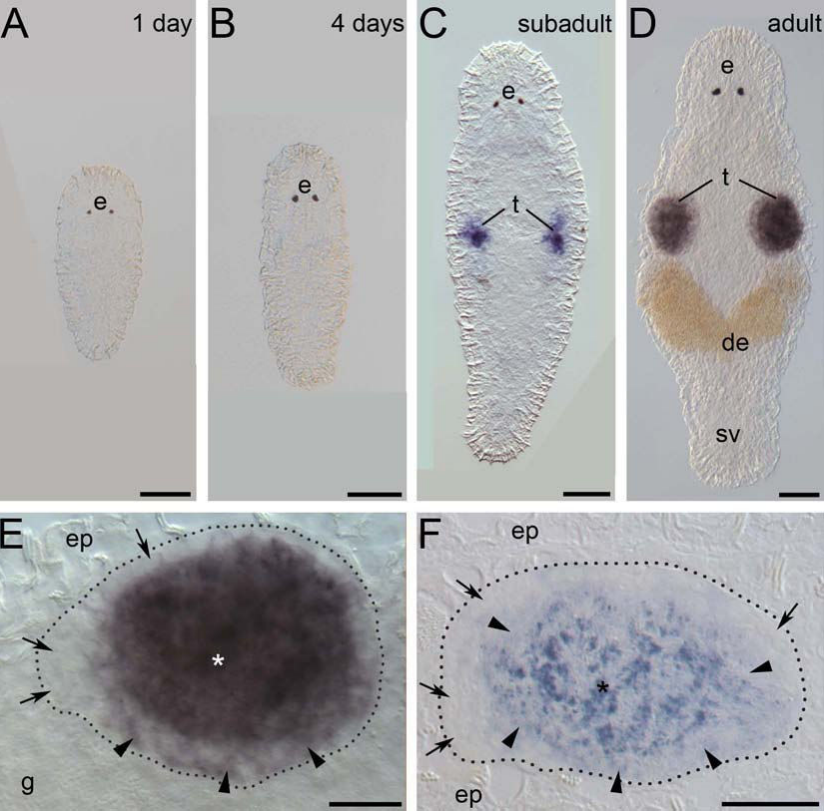
**Expression pattern of *melav2 *mRNA**. (A, B) In 1-day old hatchlings (A) and 4-days old juveniles (B) no *melav2 *signal was detected. (C) Subadult worms. The signal was present in several cells of the developing testes (t). (D) Mature adult worms. Strong expression was detected in testes (t), but not in the seminal vesicle (sv). (E) Magnified image of testis of a mature adult worm (D). Cells on the edge of testis had no or only weak *melav2 *expression (arrow and arrowhead, respectively). Strong expression was detected mainly in the centre of testis (asterisk). (F) Sagittal section of the testis after whole mount *in situ *hybridization. The head is left and the dorsal side is up. Spermatogonia, and probably also spermatocyte I were *melav2 *negative (arrow). Weak signal was detected in spermatocyte II (arrowhead) and strong expression was detected in spermatids (asterisk). e, eyes; de, developing eggs; ep, epidermis; g, gut. Scale bars: A-D, 50 μm; E, F, 20 μm.

In order to investigate the expression pattern of the *melav2 *mRNA, we performed whole mount *in situ *hybridizations (Figure 2). 1-day old hatchlings possess a gonad anlage of 4-6 primordial germ cells [6]. However, expression of *melav2 *was not yet detected at this stage (Figure 2A). *Melav2 *expression was also lacking in 4-days old worms (Figure 2B), which already have spermatogonia and spermatocytes but not yet spermatids. In subadult worms, which start to have maturing sperm, several cells in the testis were *melav2 *positive (Figure 2C). In fully mature worms, which have an increasing number of maturating sperm and also developing eggs, *melav2 *expression became strong in the testes (Figure 2D). Cells on the edge of testis had no or only weak *melav2 *expression, while the signal around the center of the testis was stronger (Figure 2E), suggesting that the expression level increased as spermatogenesis progressed. Semi-thin sectioning after whole mount *in situ *hybridization revealed that spermatogonia and probably spermatocytes I did not express *melav2 *(Figure 2F). Furthermore, it seems that a weak signal was present in spermatocytes II and a strong signal was observed in spermatids (Figure 2F). Mature sperm did not seem to express *melav2*, considering that no expression was detected in fully mature sperm within the seminal vesicle (Figure 2D). These results suggest that *melav2 *starts to be expressed at the late stage of spermatogenesis, but not anymore after spermatogenesis was completed.

### *Melav2 *RNAi causes an aberrant spermatid morphology

**Figure 3:**
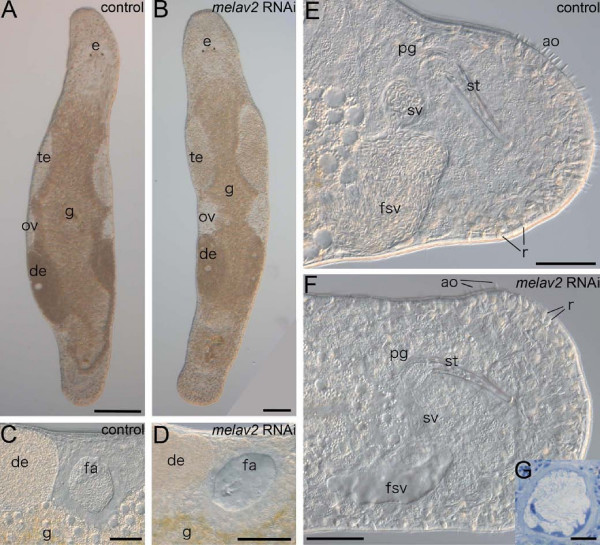
**Comparison of the overall morphology of control and *melav2 *RNAi treated *M. lignano***. (A, B) The overall of morphology of control animals (A) was comparable with that of *melav2 *RNAi treated animals (B) except for the morphology of the testis (te). Eyes (e), gut (g), ovaries (ov), developing eggs (de), and copulatory stylet (st) were not affected by the *melav2 *RNAi treatment. (C, D) The female antrum (fa) was filled with received sperm in the control animals (C), while no sperm was present in the *melav2 *RNAi treated animals (D). (E, F) In the control animals (E), the false seminal vesicle (fsv) and the seminal vesicle (sv) were filled with sperm, while no sperm was usually found there in the *melav2 *RNAi treated animals (F). The copulatory stylet (st), prostate glands (pg), rhabdites (r), gut (g) and adhesive organs (ao) were not affected by the *melav2 *RNAi treatment. Note that only a few adhesive organs are in the focal plane in this picture. (G) In a few cases, the *melav2 *RNAi treated animals had some aberrant spermatids in the seminal vesicle (G), suggesting that the vas deference was connected to the testis normally. Scale bars: A, B, 100 μm; C-F, 50 μm; G, 10 μm.

By performing RNAi gene knock-down experiments, we found that *melav2 *is essential for spermatogenesis in *M. lignano*. Apart from the differentiating spermatids, we did not find remarkable morphological defects in other tissues such as spermatogonia and spermatocytes (Additional file 1), neuropile and nerve cords (Additional file 2), epidermis, muscles and gland cells (Additional file 3), and various stages of oocyte development (Additional file 4) of *melav2 *RNAi treated worms. These observations are consistent with the testis-specific *melav2 *gene expression pattern (Figure 3). The animals had ovaries and developing eggs (Figure 3A, B), and the other reproductive organs such as the female genital opening, the antrum (Figure 3C, D), and the male copulatory stylet (Figure 3E, F) exhibited a normal phenotype. However, *melav2 *RNAi treated worms did not have any received sperm in their female antrum (Figure 3D) and had no sperm in the seminal vesicle (Figure 3F). In controls, on the contrary, both organs were full of sperm (Figure 3C, E). In a few exceptional cases (4 out of 48), we observed one or two aberrant spermatids in the seminal vesicle in the *melav2 *RNAi treated worms (Figure 3G), suggesting that the formation of the vas deferens was not affected by RNAi. We therefore conclude that the empty seminal vesicle resulted, not from a lack of a vas deferens, but from a failure of sperm maturation, as described later. *Elav *genes are known to function in the nervous system in other organisms, but the neuropile structure and the nerve cords of *melav2 *RNAi treated worms appeared unaffected (Additional file 2). Furthermore, behaviors, such as swimming or feeding, also appeared to be normal in *melav2 *RNAi treated worms.

**Figure 4:**
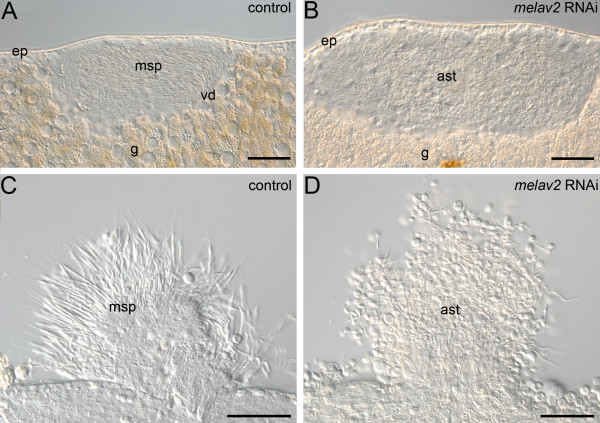
**Comparison of the testis of control and *melav2 *RNAi treated *M. lignano***. (A, B) The testis of control animals (A) had maturating sperm (msp) that were elongated and well organized towards the vas deferens (vd), while the inside of the testis in *melav2 *RNAi treated animals (B) looked disorganized and contained aberrant spermatids (ast). (C, D) Testis components from squeezed animals. In the control animals (C), many elongated maturating sperm (msp) were observed, while only aberrant spermatids (ast) were observed in the *melav2 *RNAi treated animals (D). g, gut; ep, epidermis. Scale bars: 50 μm.

The testes of *melav2 *RNAi treated worms showed severe defects (Figure 4). The testis of control worms contained many maturating sperm, which were elongated and well organized in the central region of the testis (Figure 4A). In live control animals the sperm were moving vigorously inside the testis. In the *melav2 *RNAi treated worms, however, the content of the testis looked strongly disorganized (Figure 4B), and internal movements occurred rarely. Furthermore, testis components of control animals contained normal differentiating sperm (Figure 4C), while in the *melav2 *RNAi treated worms an accumulation of aberrant cells was present (Figure 4D). Note that not many mature sperm can be seen in control worms because they are transferred to the seminal vesicle as they complete spermatogenesis.

**Figure 5:**
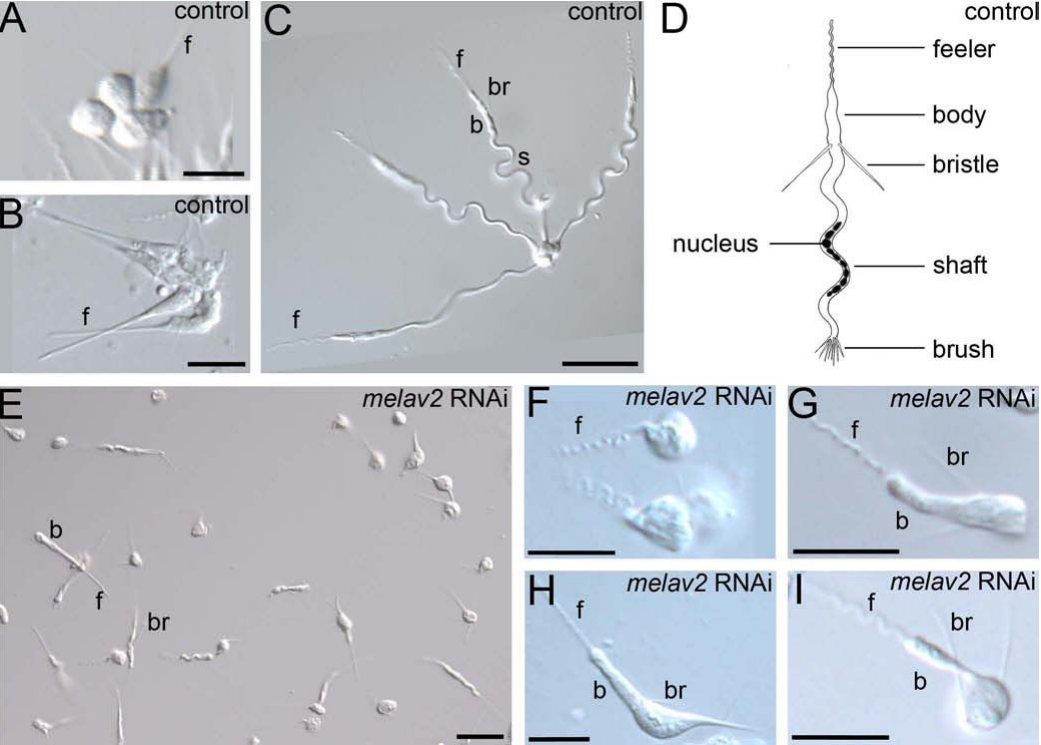
**Comparison of the sperm morphology of control and *melav2 *RNAi treated *M. lignano***. (A-C) Normal spermatid differentiation starts with the development of the anterior part. Note that spermatid cells are arranged in clusters of four cells until just before the completion of sperm maturation. (D) Schematic illustration of normal mature sperm. Note that the direction of bristles is toward posterior in mature sperm. (E) In the *melav2 *RNAi treated animals no clusters of four cells were found. (F-I) Examples of aberrant spermatid morphology in the *melav2 *RNAi treated animals. Note that the posterior part of the developing spermatids was affected by the *melav2 *RNAi treatment in all cases. b, body; br, bristles; f, feeler; s, shaft. Scale bars: A, B, F-I, 10 μm; C, E, 20 μm.

We next analyzed the morphology of single cells in detail (Figure 5). In control worms, spermatid cells were often seen as clusters of four cells until they finished spermiogenesis (Figure 5A-C). Spermiogenesis begins with the development of the feeler, then the body (Figure 5A, B) and the bristles, followed by the formation of the shaft (Figure 5C). After the the split of the tetrad the brush is formed (Figure 5D). In *melav2 *RNAi treated worms, however, most of cells were not in four-cell clusters, even before elongation was completed (Figure 5E). In addition, many spermatids showed an aberrant morphology (Figure 5F-I). In some cases, for example, round immature cells had feelers with the wavy morphology characteristic of developed sperm (Figure 5F), while the feeler of normal spermatids in controls remained straight at that stage of development (Figure 5A, B). In other cases, spermatids of *melav2 *RNAi treated animals had a feeler and a pair of bristles but failed to elongate the shaft (Figure 5G-I). This aberrant morphology was never observed in controls. Taking all these features together, the morphological defects induced by *melav2 *RNAi occurred mainly in the posterior parts, which are formed in the later stages of spermiogenesis.

### *Melav2 *is necessary for spermiogenesis

**Figure 6:**
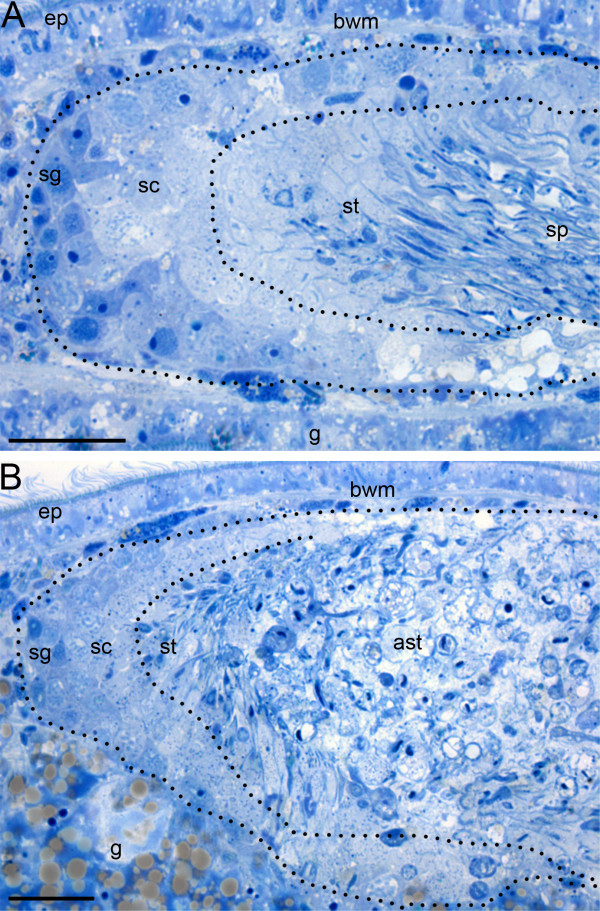
**Comparison of the spermatogenesis of control and *melav2 *RNAi treated *M. lignano***. In the control animals (A), spermatogonia (sg) and spermatocytes (sc) were observed in the peripheral region of the testis, and elongating spermatids (st) and sperm (sp) were present in the center. In the *melav2 *RNAi treated animals (B), spermatogonia (sg) and spermatocytes (sc) were also observed to be of normal morphology, but the center region of the testis was filled with aberrant spermatids (ast) with large vacuoles. Dotted lines roughly indicate the regions that contain the respective cell types. g, gut; ep, epidermis; bwm, body wall musculature. Scale bars: 20 μm.

In order to determine exactly which stage of spermatogenesis was disturbed by *melav2 *RNAi, we compared semi-thin sections of control and *melav2 *RNAi treated worms. We found that spermatogenesis started to fail at the spermatid stage in the *melav2 *RNAi treated worms (Figure 6). In the testes of control worms (Figure 6A), spermatogonia and spermatocytes were observed at the peripheral regions, whereas elongating spermatids and sperm were located at the center. In the *melav2 *RNAi treated worms (Figure 6B), spermatogonia and spermatocytes seemed likely to be normal (Additional file 1). However, from the spermatid stage onwards, cells started to become aberrant. The center of the testis was full of these aberrant cells with large vacuoles (Figure 6B).

**Figure 7:**
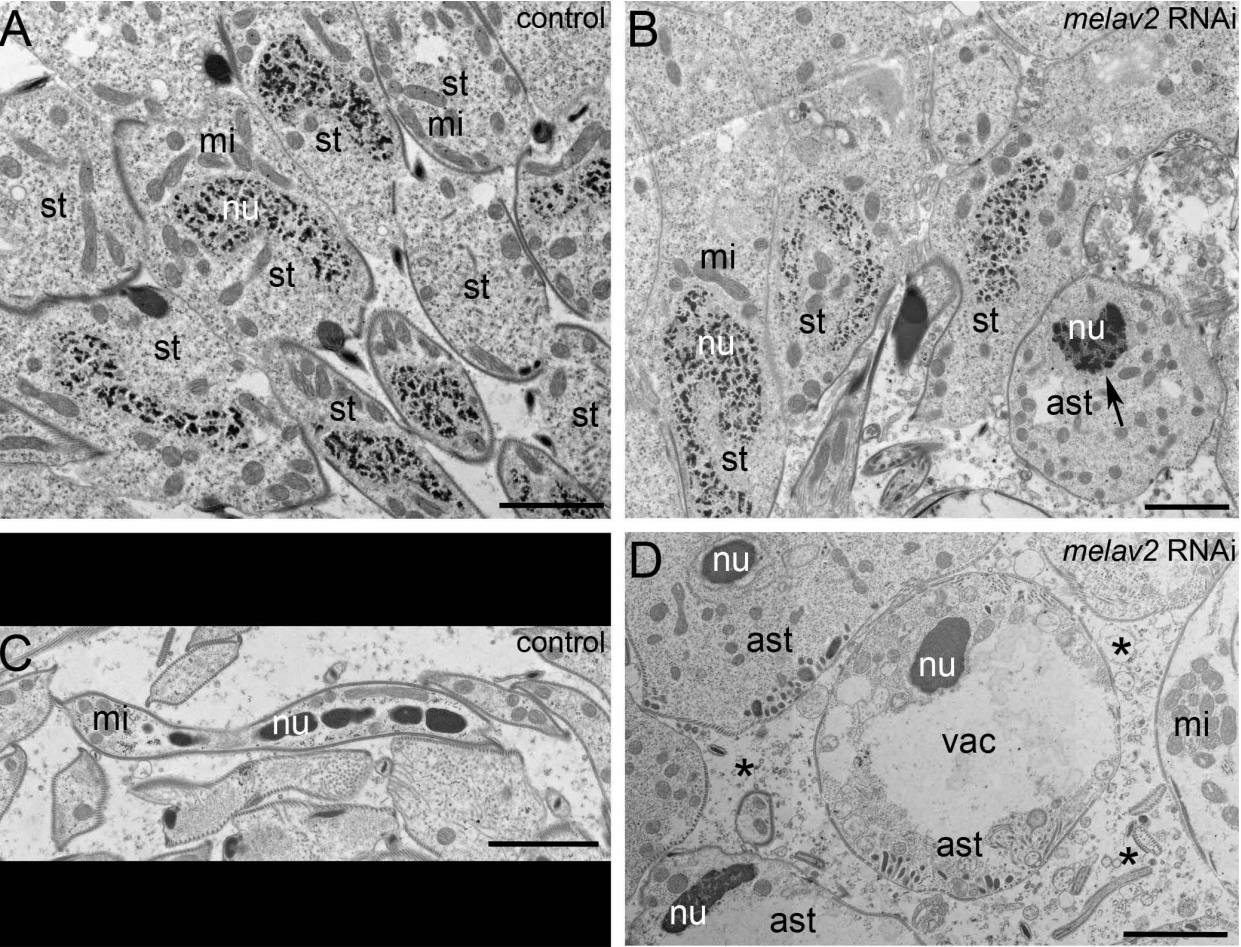
**Comparison of spermatid differentiation of control and *melav2 *RNAi treated *M. lignano *by TEM**. (A, B) In the control animals (A), the nucleus of a spermatid was never tightly condensed until quite a late stage of spermiogenesis, while in the *melav2 *RNAi treated animals (B), some spermatids started to show a condensed nucleus precociously (B, arrow). (C, D) In the control animals (C), mature sperm had an elongated morphology and the nucleus was condensed into the train-shape, while in the *melav2 *RNAi treated animals (D), the train-shaped nuclei were never observed, and the cells often had large vacuoles (vac). Some cell components were also observed outside of the cells (D, asterisk), suggesting cells were dying by necrosis. st, spermatid; ast, aberrant spermatid; mi, mitochondria; nu, nuclei. Scale bars: 2 μm.

Transmission electron microscopy revealed a failure of nucleus reorganization between the middle and the late stages of spermatid development (Figure 7). First, spermatogonia and spermatocytes I and II looked normal in both control and *melav2 *RNAi treated worms (Additional file 1). In control worms (Figure 7A), chromatin started to condense gradually during spermatid elongation and became reorganized into the train-shaped morphology in later stages (Figure 7C). In the *melav2 *RNAi treated worms, the early stage of spermatid development appeared to be normal, except for a precocious condensation of the chromatin (Figure 7B). Around the middle stages of spermatid development, we often observed already tightly condensed patches of chromatin (Figure 7D), which was never found in the control worms. In addition, these cells often had large vacuoles in the cytoplasm (Figure 7D and also Figure 6B). In some cases, cell components such as mitochondria and other organelles were observed outside of cell (Figure 7D), suggesting that cells were dying by necrosis. Cells with train-shaped nuclei were never observed in the *melav2 *RNAi treated worms.

### *Melav2 *RNAi interrupts the transition from spermatid to sperm

**Figure 8:**
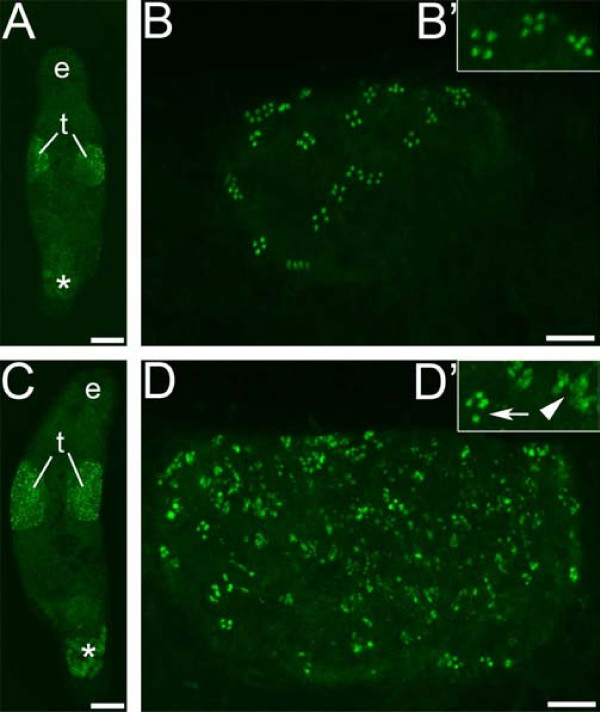
**MSp-1 antibody staining for early spermatid cells in control and *melav2 *RNAi treated *M. lignano***. The testis of control animals (A overview, B detail) had normal MSp-1 signal present in clusters of four cells (B, B'), while the testis of *melav2 *RNAi treated animals (C overview, D detail) had a considerably larger number of MSp-1 positive cells (D, D'). Note that the *melav2 *RNAi treated animals had some normal MSp-1 signal (D' arrow), but also a lot of disorganized signal (D' arrowhead). e, eyes; t, testes. Asterisk indicates non-specific signal. Scale bars: A, C, 100 μm; B, D, 20 μm.

Immunocytochemical staining with the monoclonal MSp-1 antibody, which recognizes only early spermatids in *M. lignano *[7], revealed that many cells did not complete the transition from early spermatid stage properly in *melav2 *RNAi treated worms (Figure 8). In control worms the signal was always detected in clusters of four cells and the number of MSp-1-positive cells did not exceed a few dozens (Figure 8A, B). In the *melav2 *RNAi treated worms, however, a considerably larger amount of MSp-1-positive cells was observed (Figure 8C, D), suggesting an accumulation of these cells in the testis. In some cases, the signal looked normal and cells were present in a cluster of four cells, but most of the MSp-1 signal looked disorganized (Figure 8D'). These results suggest that the spermatid differentiation until early spermatid stage was normal but the transition to a later spermatid stage was not completed properly. Therefore many later spermatids remained to hold a certain level of Msp-1 protein in an aberrant appearance, probably because they were partially arrested, differentiating and/or dying.

## Discussion

### *Melav2 *encodes for an *elav*-like gene

Our results clearly show a new biological function of an *elav*-like gene, namely the involvement of *melav2 *in spermatid differentiation in *M. lignano*. *Elav *family genes have been mainly described to be involved in neural processes, such as the transition between proliferation and differentiation, maturation, maintenance of neurons, and learning [20,26,32-34] ([35]. Recently, however, also other biological functions have been reported. For example, RBP9, a *Drosophila *paralog of *elav*, is expressed during oogenesis and is required for female fertility [36]. EXC-7, a *C. elegans *ELAV homologue, is involved in the developent of excretory canals [37]. And some *elav *family members are expressed in other tissues or ubiquitously [38], suggesting non-neuronal functions. In our study, we did not find remarkable effects of *melav2 *RNAi on the nervous system, although we cannot exclude the possibility of *melav2 *functions in neural cells in pre-hatching stages. However, we found another *elav*-like gene in *M. lignano*, *melav1 *(Figure 1). As previously outlined, the focus of the current study was on the testis-specific expression of *melav2*, and we therefore did not explore the function of *melav1*. We speculate that it may have a role in nervous system similar to other organism's *elav*-like genes (particularly given that it appears to more closely resemble these genes, Figure 1C).

*Elav *genes are characterized by three RRMs with a hinge region between the second and the third RRM. RRM is a quite common protein domain in eukaryotes [22,23,25]. For example, a recent analysis of ESTs in the planarian *Schmidtea mediterranea *revealed that RRM was the second most common domain [39]. It is considered that slight changes in RRM can lead to different interactions with other proteins, leading to various types of functions [23]. In fact, recent findings revealed diverse molecular functions for members of the *elav *gene family. For example, *elav *has been shown to regulate alternative splicing of *neuroglian, erect wing *and *armadillo *transcripts in *Drosophila *[40-42]. The human *elav *gene Hel-N1 (HuB) has been suggested to stabilize *GLUT1 *mRNA and to increase its translational efficiency [43]. In *melav2*, the similarity of the third RRM was not well conserved compared to the first and the second RRM. Recently, it has been indicated that the Elav protein can form multimeric complexes [44,45], and *in vitro *experiments suggested that the third RRM has a role for the multimerization, although this is not its only function [46]. It might be possible that the difference in the third RRM of *melav2 *contributes to a new interaction with the target mRNAs involved in spermatogenesis.

### *Melav2 *is necessary for spermatid differentiation

The *melav2 *RNAi phenotype showed severe defects in spermatids and an abnormally condensed chromatin. We do not consider these features as signs of an apoptotic process, but instead we found several indications for necrosis. First, cell components such as mitochondria were observed outside of cells (Figure 7D), suggesting that cells were dying in a necrotic way. Second, we observed neither nuclear fragmentation nor an apoptotic body, which are typical characteristics of apoptosis also in *M. lignano *[47]. Third, cells often showed cytoplasmic vacuolization (Figure 7D), which is unusual for apoptosis. Fourth, we observed an accumulation of cells that failed to proceed properly into the later spermatid stages (Figure 8B), suggesting that the elimination of such failed cells by programmed cell death did not occur.

We propose two possible explanations for the abnormal chromatin condensation and the failure of spermatid elongation. One possibility is that *melav2 *regulates the genes that mediate chromatin condensation, and chromatin condensation occurred too early as a result of *melav2 *RNAi, leading to cessation of further sperm maturation, possibly due to an early stop in the transcription of essential genes. A similar phenotype was shown in spermiogenesis of transgenic mice where pre-mature translation of *prm-1 *occurred [48]. *Prm-1 *should be repressed until a later spermatid stage for a proper transition from nucleohistones to nucleo-protamines. Premature translation of *prm-1 *caused early chromatin condensation in round spermatids, a subsequent arrest in spermiogenesis and an aberrant spermatid morphology. These features correspond well with the *melav2 *RNAi phenotype shown here (Figure 7, 8 and 5, respectively) and therefore it appears possible that *melav2 *has a function in regulating *prm-1 *or other comparable genes.

Another possible explanation is that cells retained their round shape because of the disruption of spermatid elongation. It has been reported that a human neuronal Elav protein, Hel-N1 (HuB) gene, upregulates the translation of neurofilament M (NF-M) mRNA by using human embryonic teratocarcinoma cells (hNT2) transfected with Hel-N1 [49]. Overexpressed Hel-N1 did not affect the level of NF-M mRNA expression but instead recruited it into heavy polysomes more efficiently, resulting in the formation of neurites. If a similar molecular function is performed by *melav2*, it is possible that *melav2 *is involved in promoting translation of some cytoskeletal elements such as sperm-specific intermediate filaments. The shortage of these elements caused by *melav2 *RNAi could result in cessation of elongation, as is often seen in the posterior part at later spermatid stage (Figure 5F-I) and the failed chromatin reorganization, in which the chromatin was condensed but never built in a train shape (Figure 7D).

Although *melav2 *has three RRMs, we have no direct evidence whether *melav2 *really functions as RNA-binding protein and, if so, what kinds of mRNA are targeted. Thus, identifying the target genes of *melav2 *and their interaction might be important follow-up experiments. Recently, a transcriptome and a genome sequencing project have been initiated in *M. lignano*. Therefore it will soon be possible to identify *melav2 *downstream genes by microarrays or *in silico *comparison of transcriptomes of control animals with *melav2 *RNAi treated worms.

### *Melav2 *RNAi treated worms are male-sterile

The seminal vesicles of *melav2 *RNAi treated worms were empty, and in consequence they cannot transfer sperm to a mating partner, suggesting they are male-sterile. However, *M. lignano *is hermaphroditic and the female reproductive function of *melav2 *RNAi treated worms was normal (Figure 3). So a *melav2 *RNAi treated animal (or a loss of function mutant) would still be able to contribute offspring to next generation as a female after copulation with normal worms. Thus, future experiments aimed at following their reproductive success of such 'females' and determining if such functional females could spread within a hermaphroditic population, would provide a better understanding of the evolution of different reproductive modes. In addition, the formation of the stylet and the male accessory glands in *melav2 *RNAi treated worms was normal (Figure 3), which offers interesting possibilities for studies on sexual conflicts. Recent experiments have suggested that male accessory glands and their secretions have important roles for increasing male reproductive success by manipulating the female reproductive physiology and behavior, such as increasing egg production, decreasing female's motivation toward further copulations with other partners, and decreasing female's longevity [50-52]. Although we still need to analyze if *melav2 *RNAi treated worms can in fact transfer ejaculates without sperm, such worms have a great potential to study the effects of seminal fluids in hermaphroditic organisms, by removing the effect of the transferred sperm itself.

## Conclusion

We found that *melav2 *has a similarity with the *elav *gene family at the protein sequence level. *Elav *genes are mainly known to be involved in the nervous system in other organisms, but our study clearly shows that *melav2 *plays a crucial role during spermatid differentiation in *M. lignano*. *Melav2 *RNAi disturbed the proper regulation of chromatin condensation and/or the cell elongation, resulting in aberrant spermatid morphology and male sterility.

## Methods

### Animal culture

The free-living flatworm *M. lignano *[14] was cultured in glass Petri dishes with nutrient-enriched artificial seawater (Guillard's f/2 medium [53]) and fed with the diatom *Nitzschia curvilineata*. The worms were kept in a climate chamber with 60% humidity at 20°C in a 14:10 h day-night cycle [54]. Animal experimentation was carried out in accordance to Austrian legal and ethical standards.

### Gene isolation and analyses

The full length open reading frame of the clone ANGU919 http://flatworm.uibk.ac.at/macest/ was sequenced by GATC (Konstanz, Germany) using M13 standard primers. Sequences were analyzed by BLASTX searches at the EMBL-EBI database http://www.ebi.ac.uk/Tools/blast2/. Conserved protein domains were identified using the SMART databases http://smart.embl-heidelberg.de/[55]. Protein structural analysis was performed using the SWISS-MODEL program http://swissmodel.expasy.org/[56,57].

### Alignment and phylogenetic analyses

Amino acid alignments were performed using the Multiple Sequence Alignment Program CLUSTALW of the EMBL-EBI database http://www.ebi.ac.uk/Tools/clustalw2/ with default alignment parameters. The phylogenetic tree was calculated using the MrBayes 3.2.2 [58,59]. Each run was performed using default parameters and comprised 5,000,000 generations.

Elav sequences were from *Mus musculus *ELAV-like protein1 [UniProtKB:P70372], *Xenopus laevis *ELAV-like2 [GenBank:NP_001081035], *Danio rerio *ELAV-like 1 [GenBank:NP_571527], *Drosophila melanogaster *Protein elav [UniProtKB:P16914], *Aplysia californica *ELAV 2-like protein [GenBank:AAY42042], *Platynereis dumerilii *Elav [GenBank:ABO93208]. Elav sequences of *Schmidtea mediterranea *and *Hydra magnipapillata *were generated by computational work using available EST database, SmedGD v1.3.14 http://khan.neuro.utah.edu/index.html and dbEST_HMAG070214 http://www.compagen.org, respectively. The *Schmidtea *sequence was generated from contig ec1.03596.005, and the *Hydra *sequence was generated by assembling three clones, tai96e09.y2 [CX056199], tai87h08.y1 [CV182751] and tai87h08.x1 [CV182482]. *Melav1*, another *elav*-like gene from *M. lignano *was obtained from EST clone Ml_aw_006_D03 in a same way as the *melav2 *gene.

Sex lethal sequences were from *Drosophila melanogaster *Protein sex-lethal [UniProtKB:P19339], *Drosophila subobscura *Protein sex-lethal [UniProtKB:Q24668], *Musca domestica *Sex-lethal homolog [UniProtKB:O17310], *Ceratitis capitata *Sex-lethal homolog [UniProtKB:O61374], *Chrysomya rufifacies *Sex-lethal homolog [UniProtKB:O97018], *Megaselia scalaris *Sex-lethal homolog [UniProtKB:O01671], *Anopheles gambiae *TPA:sex-lethal [GenBank:CAJ55784], *Acyrthosiphon pisum *sex-lethal [GenBank:NP_001119609], *Bombyx mori *sex-lethal [GenBank:NP_001036780]. As an outgroup for the calculation of phylogeny, *Drosophila melanogaster *CG4787-PA [UniProtKB:Q9VAX1] was used. Square brackets indicate accession number.

### Whole mount *in situ *hybridization

The sequence region for the *in situ *RNA probe was amplified with the *melav2 *specific primers 5'-GGC CTT CTC AGA TGA CGA GT-3' and 5'-GGA CAG ATG TTG ATG GAC CTG-3'. The PCR condition was 2 min at 94°C, 35 cycles (30 sec at 94°C, 30 sec 55°C, 90 sec at 72°C), 7 min at 72°C. Obtained PCR products were sub-cloned into pGEM^®^-T (Promega). Then PCR with M13 standard primers was performed to generate the template for RNA probe synthesis, including SP6 and T7 RNA polymerase promoter sequences. Digoxygenin-labeled RNA probe was generated using DIG RNA Labeling KIT SP6/T7 (Roche), following the manufactor's protocol. Whole mount *in situ *hybridization for *M. lignano *was performed as previously described [6]. The signal was developed at 37°C using the NBT/BCIP system (Roche). Specimens were examined with a Leica DM5000 microscope. Image acquisition and analysis were performed using a Leica DFC490 digital camera, the Leica Application Suite 2.8.1 software, and the Adobe^® ^Photoshop^® ^7.0 software.

### RNA interference

By adding T7 RNA polymerase promoter sequence to the primers that were used to generate the templates for *in situ *RNA probe, two different templates for dsRNA probe were generated: one had a T7 promoter at the 5' end for producing sense RNA, and the other had it at the 3' end for producing anti-sense RNA, respectively. The PCR condition was 2 min at 94°C, 35 cycles (30 sec at 94°C, 30 sec 55°C, 90 sec at 72°C), 7 min at 72°C. The *in vitro *synthesis of dsRNA was performed using T7 RiboMax TM Express RNAi System (Promega). As a negative control, firefly *luciferase *dsRNA was produced from the pGEM^®^-luc Vector (Promega). RNAi treatment for *M. lignano *was performed by soaking as previously described [6]. We used 31 animals for control and 55 animals for *melav2 *RNAi treatment. One-day old hatchlings were maintained in 24-well plates (20 worms per well) and incubated with 250 μl dsRNA solution (3.0 ng/μl) in f/2 medium containing the antibiotics Kanamycin and Ampicillin (50 μg/ml, respectively) and diatoms. dsRNA solution was changed twice a day. After 3-4 weeks of RNAi treatment, the specimens were examined with a Leica DM5000 microscope using DIC optics. Image acquisition and analysis were performed using the same imaging set-up as described in the "Whole mount *in situ *hybridization" section. For the observation of testis components and sperm morphology, live worms were squeezed under a cover slip until the testis ruptured and the contents of the testis were directly observed.

### Histology

Control and *melav2 *RNAi treated animals were relaxed in 7.14% MgCl_2 _and fixed for 1 hour at 4°C in 2.5% glutardaldehyde in 0.1 M cacodylate buffer (pH 7.4) containing 9% sucrose. After several washes with buffer, specimens were postfixed in 1% osmium tetroxide in 0.05 M cacodylate puffer (pH 7.4) for 1 hour. After washing in buffer and subsequent dehydration in an ethanol series, animals were embedded in SPURR"s low viscosity resin [60]. Complete series of 1.5 μm thick semi-thin sections were cut with a prototype of a Butler diamond knife (Diatome) and mounted on glass slides. After drying the sections were stained for 2 minutes in a methylen blue Azur II mixture after Richardson [61] and mounted in cedar wood oil. One control animal and one *melav2 *RNAi treated animal were examined with a Leica 5000B microscope. Image acquisition and analysis were performed using the same imaging set-up as described in the "Whole mount *in situ *hybridization" section.

### Histology after whole mount *in situ *hybridization

After performing *melav2 *whole mount *in situ *hybridizations as described above, specimens were fixed with BOUIN's fluid overnight. After subsequent dehydration in an ethanol series, animals were embedded in SPURR's low viscosity resin. Complete series of 2 μm thick semi-thin sections were cut with the same sectioning set-up as described in the "Histology" section. Two animals were examined with a Leica 5000B microscope. Image acquisition and analysis were performed using the same imaging set-up as described in the "Whole mount *in situ *hybridization" section.

### Transmission electron microscopy

Control and *melav2 *RNAi treated animals were fixed and embedded as described above. Semi-thin and ultra-thin sections were cut with a diamond knife on an Ultracut S (Leica) ultramicrotome, double stained with uranyl acetate and lead citrate. One control animal and one *melav2 *RNAi treated animal were examined with a ZEISS Libra 120 energy filter electron microscope. Image acquisition and analysis were performed using a 2 k Vario Speed SSCCD camera (Droendle) and the iTEM software (TEM imaging platform, Olympus).

### Immunocytochemistry

Immunocytochemistry for *M. lignano *was performed as previously described [6,7]. As the primary antibody, the spermatid-specific mouse monoclonal MSp-1 antibody [7] was used (1:200). As the secondary antibody, a FITC-conjugated goat-anti-mouse antibody (1:250, DAKO) was used. Two control animals and three *melav2 *RNAi treated animals were examined with a Zeiss LSM 510 confocal laser scanning microscope system. Image acquisition and analysis were performed using the LSM510 ver.3.2 software, the LSM image browser ver.4.2.0.121 software and the Adobe^® ^Photoshop^® ^7.0 software.

## Abbreviations

RNAi: RNA interference; RRM: RNA recognition motif.

## Authors' contributions

KS performed all experimental aspects of the study except the histological sections, and wrote the manuscript. WS made the histological sections and assisted KS with the histological analysis. KDM assisted KS with all experimental methods except the histology. LS and PL conceived the project and participated in the preparation of the manuscript. PL supervised the experimental work. All authors read and approved the final manuscript.

## Supplementary Material

Additional file 1 **Comparison of early spermatogenesis of control and *melav2 *RNAi treated *M. lignano *by TEM**. (A-F) The appearance of the spermatogonia and spermatocytes I and II of the control animals (A, C, D, respectively) was comparable to that of the *melav2 *RNAi treated animals (B, D, F, respectively). Scale bars: 2 μm. Click here for file

Additional file 2 **Comparison of the neuropile and nerve cord morphology of control and *melav2 *RNAi treated *M. lignano***. (A, B) The appearance of the neuropile of control animals (A) was comparable to that of the *melav2 *RNAi treated animals (B) in interference contrast microscopy. (C, D) The tissue structure of the neuropile of control animals (C) was also comparable to that of the *melav2 *RNAi treated animals (D) in semi-thin sections. Dotted lines roughly indicate the regions of the neuropile. (E, F) Morphology of nerve cord of control (E) was compatible to that of the *melav2 *RNAi treated animals (F). e, eye; ep, epidermis; gl, gland; mo, mouth opening; mu, muscle; nc, nerve cord; np, neuropile. Scale bars: A, B, 50 μm; C, D, 25 μm; E, F 2 μm. Click here for file

Additional file 3 **Comparison of tissue organization, epidermal-, muscle, and gut cell morphology of control and *melav2 *RNAi treated *M. lignano***. Overview demonstrates that tissue integrity is comparable in control (A) and *melav2 *RNAi treated (B) *M. lignano*. Likewise, the ultrastructure of epidermal cells (C, D) and gut cells (E, F) was not affected by *melav2 *RNAi treatment. c, cilia of gut cell; cm, circular mucscle; enc, epidermal cell nucleus; ep, epidermal cell; epc, epidermal cell cilia; gc, gland cell; mu, muscle cell; mv, microvilli; lm, longitudinal muscle; uhr, ultrarhabdites; v, storage vesicle. Scale bars: A, B, 10 μm; C-F, 2 μm. Click here for file

Additional file 4 **Comparison of oogenesis of control and *melav2 *RNAi treated *M. lignano***. The oocyte of control (A) and *melav2 *RNAi treated (B) *M. lignano *exhibited comparable morphology. The ultrastructure of developing eggs (C, D) egg granules (E, F) was not affected by *melav2 *RNAi treatment. de, developing egg; egr, egg granules; nl, nucleolus; onu, oocyte nucleus. Scale bars: A-D 2 μm; E, F, 1 μm. Click here for file
