# Supplementary figures and images for: Melav2, an elav-like gene, is essential for spermatid differentiation in the flatworm Macrostomum lignano
Source: BMC Dev Biol. 2009 Dec 8;9:62. doi: 10.1186/1471-213X-9-62 (PMC2795745; doi:10.1186/1471-213X-9-62)

spermatogonium

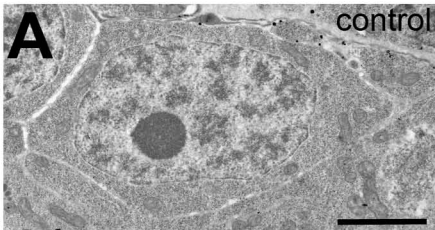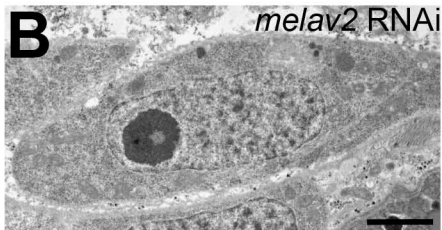

spermatocyte I

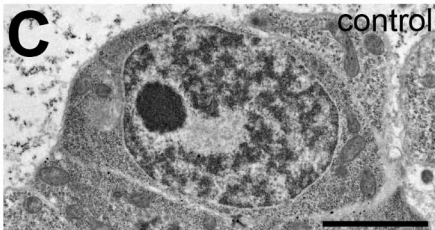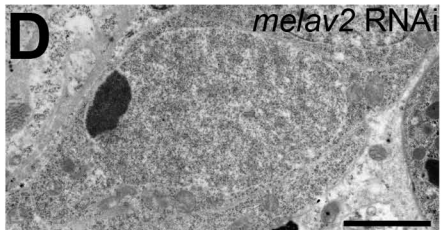

spermatocyte II

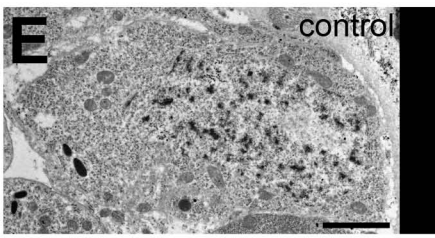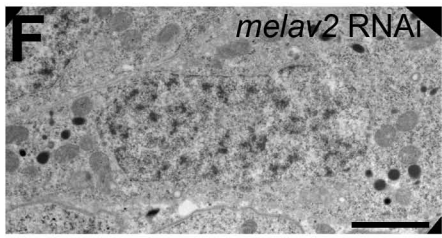

Supplement: Additional file 1 — Comparison of early spermatogenesis of control and melav2 RNAi treated M. lignano by TEM. (A-F) The appearance of the spermatogonia and spermatocytes I and II of the control animals (A, C, D, respectively) was comparable to that of the melav2 RNAi treated animals (B, D, F, respectively). Scale bars: 2 μm. [file 1471-213X-9-62-S1.PDF]

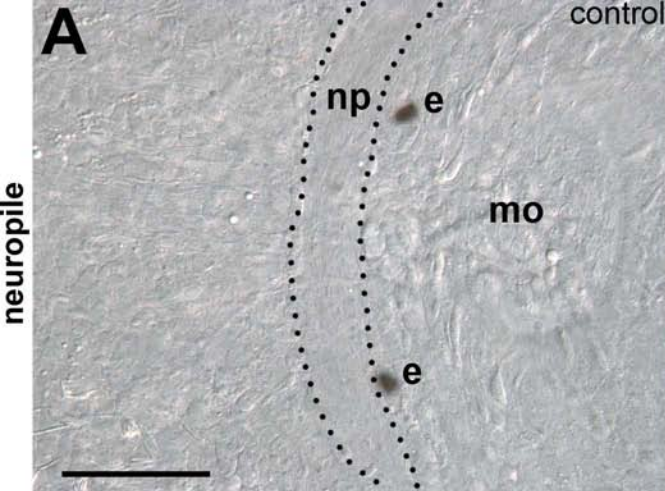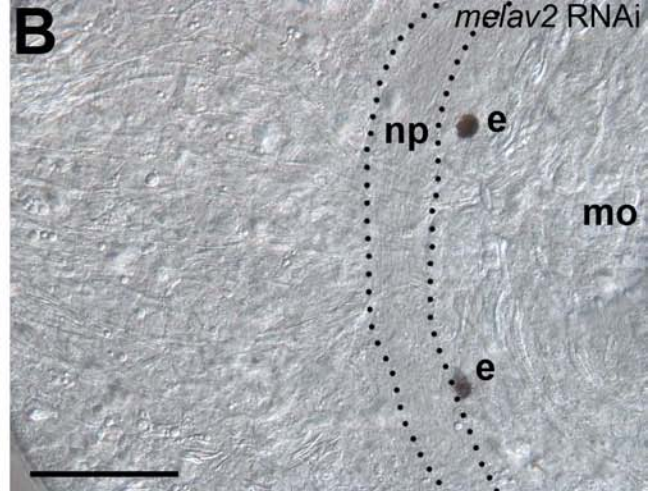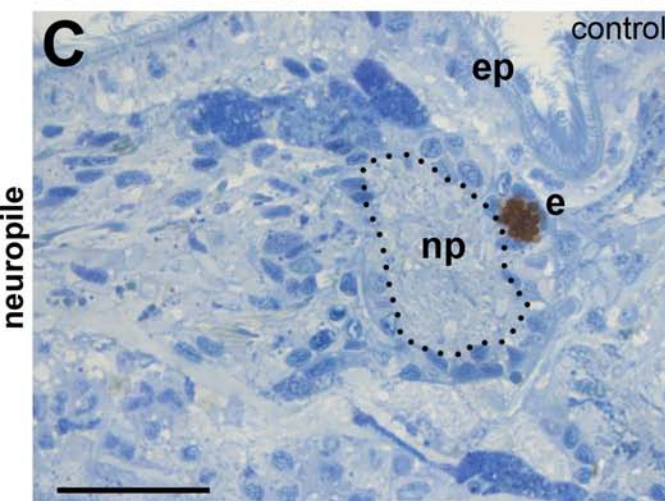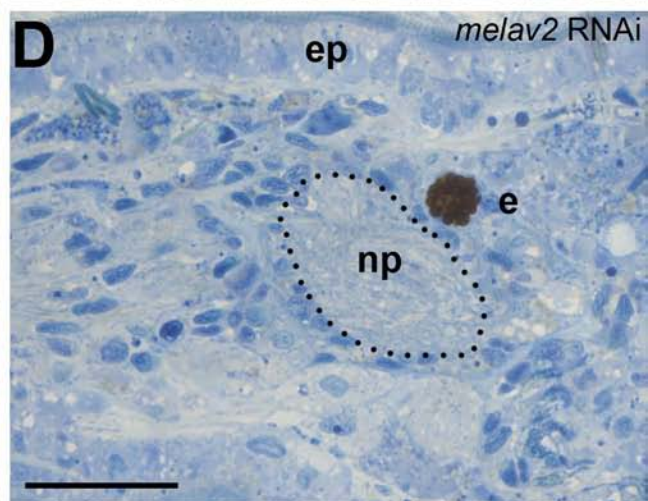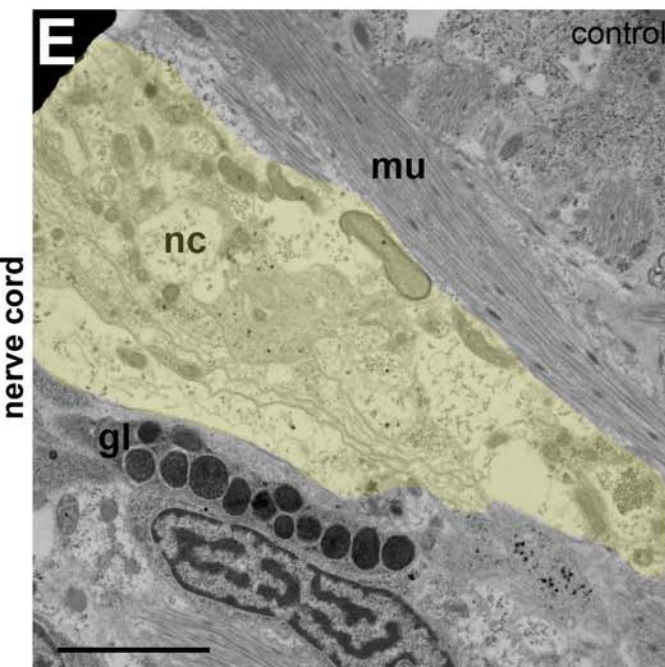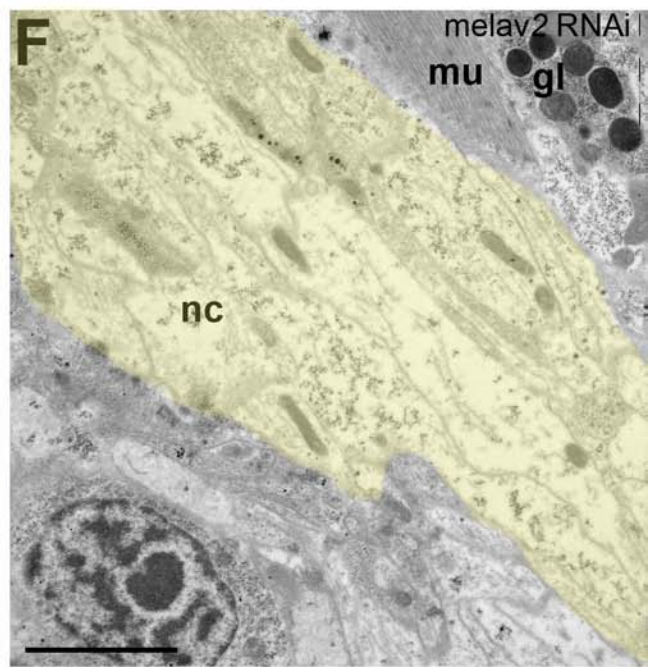

Supplement: Additional file 2 — Comparison of the neuropile and nerve cord morphology of control and melav2 RNAi treated M. lignano. (A, B) The appearance of the neuropile of control animals (A) was comparable to that of the melav2 RNAi treated animals (B) in interference contrast microscopy. (C, D) The tissue structure of the neuropile of control animals (C) was also comparable to that of the melav2 RNAi treated animals (D) in semi-thin sections. Dotted lines roughly indicate the regions of the neuropile. (E, F) Morphology of nerve cord of control (E) was compatible to that of the melav2 RNAi treated animals (F). e, eye; ep, epidermis; gl, gland; mo, mouth opening; mu, muscle; nc, nerve cord; np, neuropile. Scale bars: A, B, 50 μm; C, D, 25 μm; E, F 2 μm. [file 1471-213X-9-62-S2.PDF]

Overview

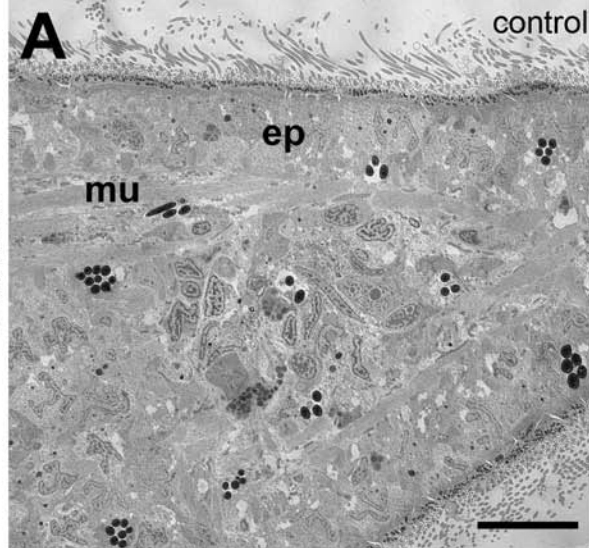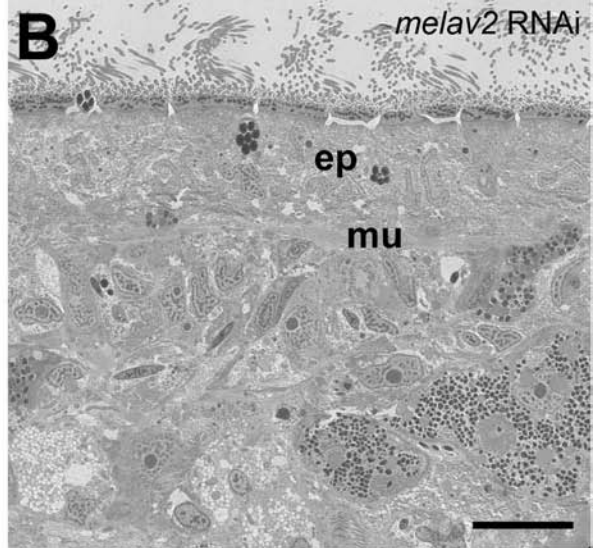

epidermis & muscle

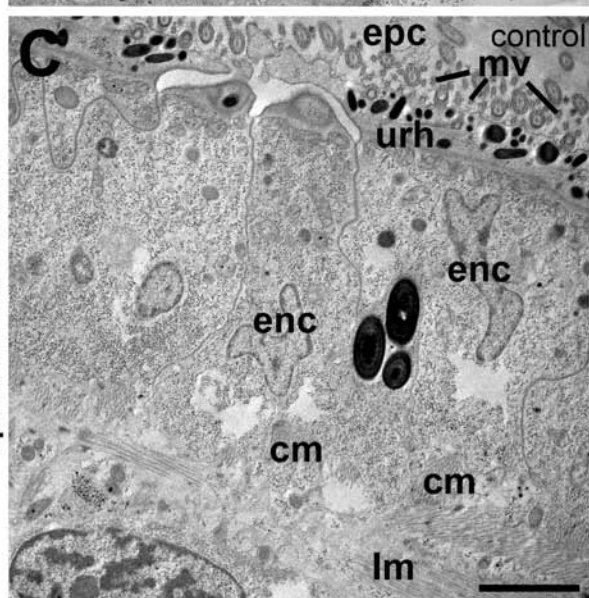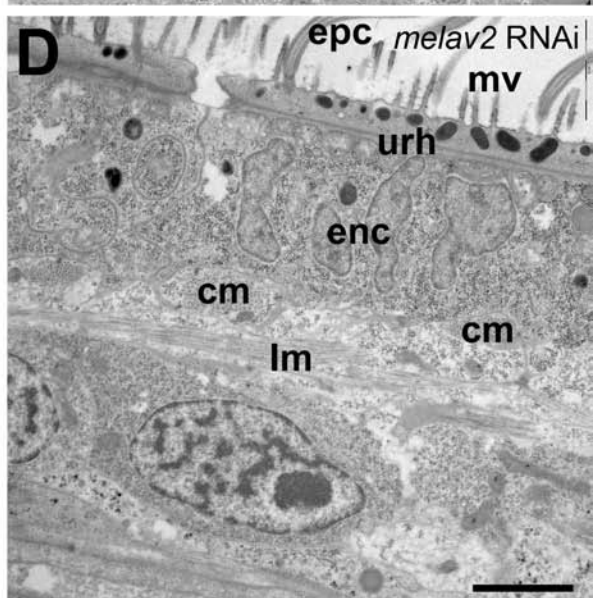

gut cell

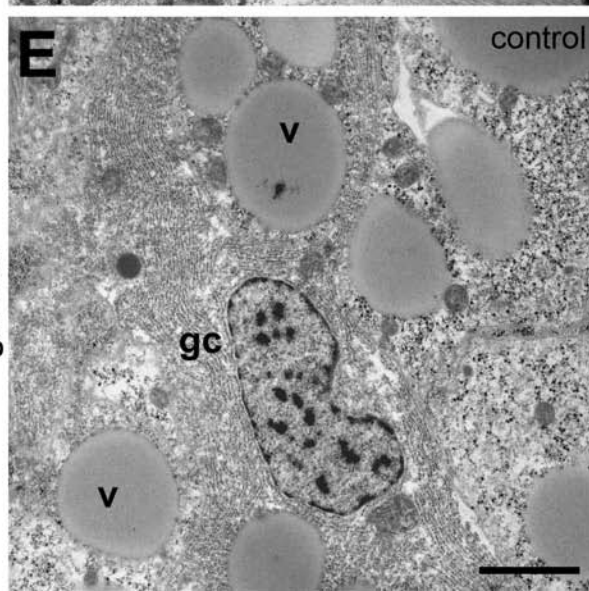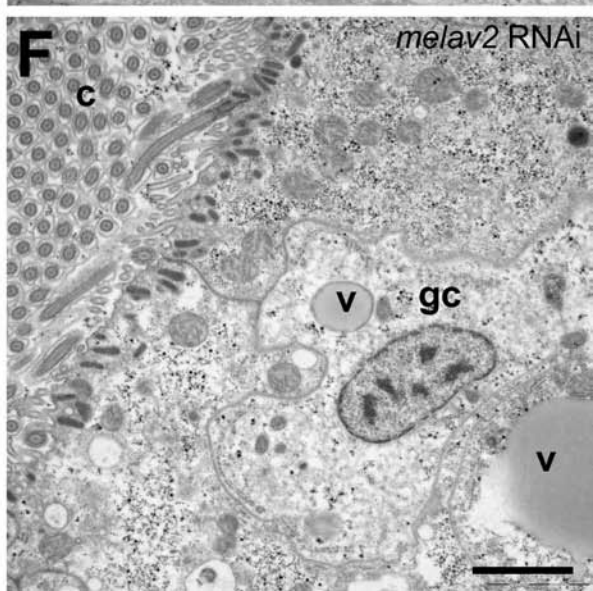

Supplement: Additional file 3 — Comparison of tissue organization, epidermal-, muscle, and gut cell morphology of control and melav2 RNAi treated M. lignano. Overview demonstrates that tissue integrity is comparable in control (A) and melav2 RNAi treated (B) M. lignano. Likewise, the ultrastructure of epidermal cells (C, D) and gut cells (E, F) was not affected by melav2 RNAi treatment. c, cilia of gut cell; cm, circular mucscle; enc, epidermal cell nucleus; ep, epidermal cell; epc, epidermal cell cilia; gc, gland cell; mu, muscle cell; mv, microvilli; lm, longitudinal muscle; uhr, ultrarhabdites; v, storage vesicle. Scale bars: A, B, 10 μm; C-F, 2 μm. [file 1471-213X-9-62-S3.PDF]

Oocyte

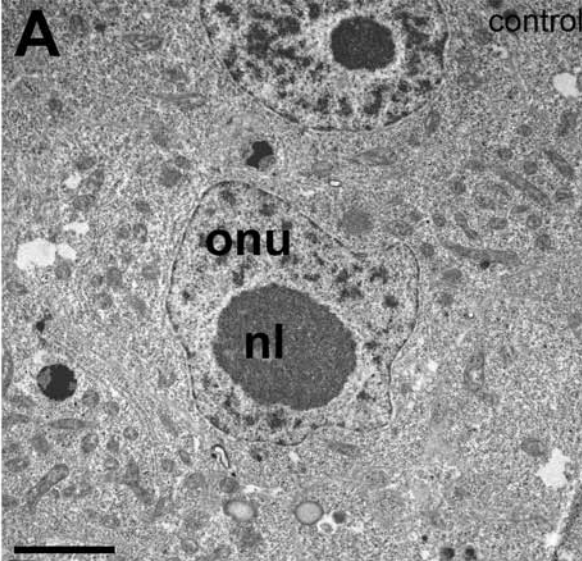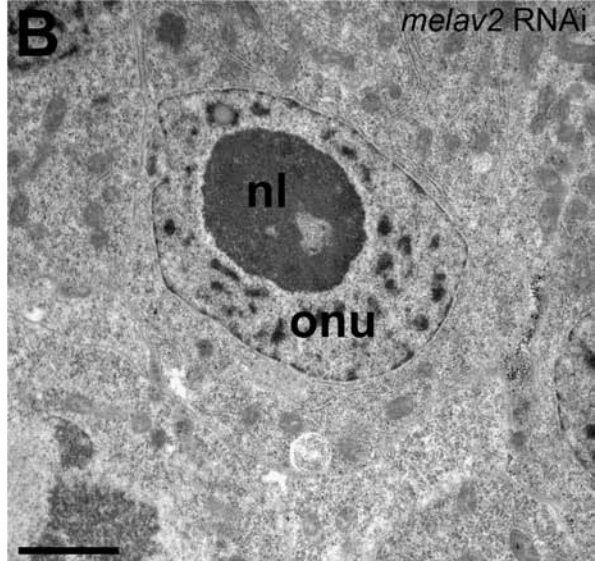

developing egg

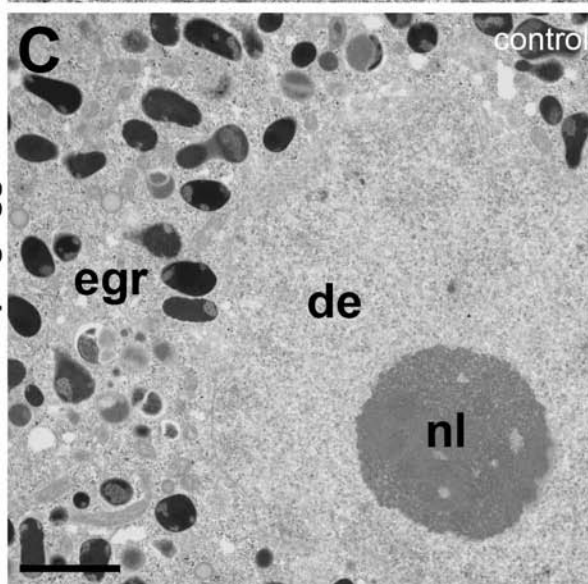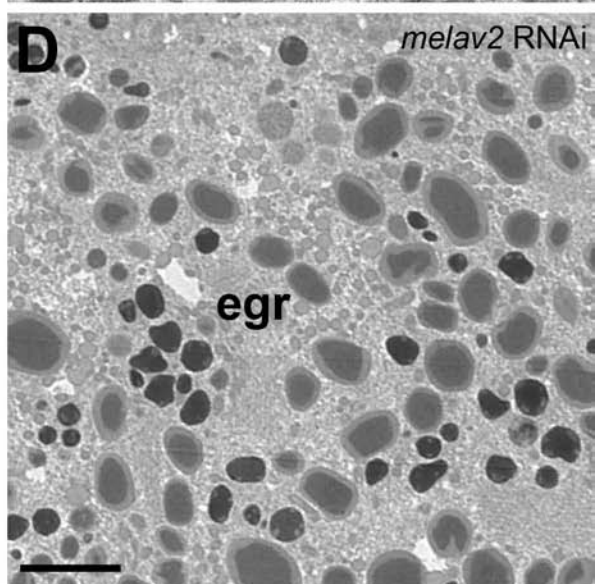

egg granules

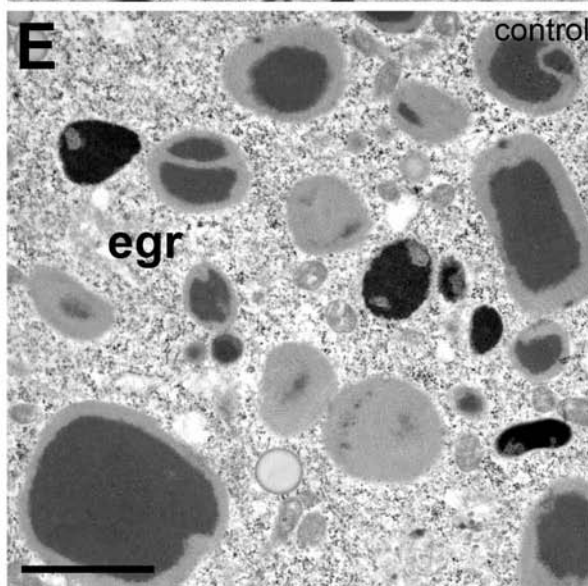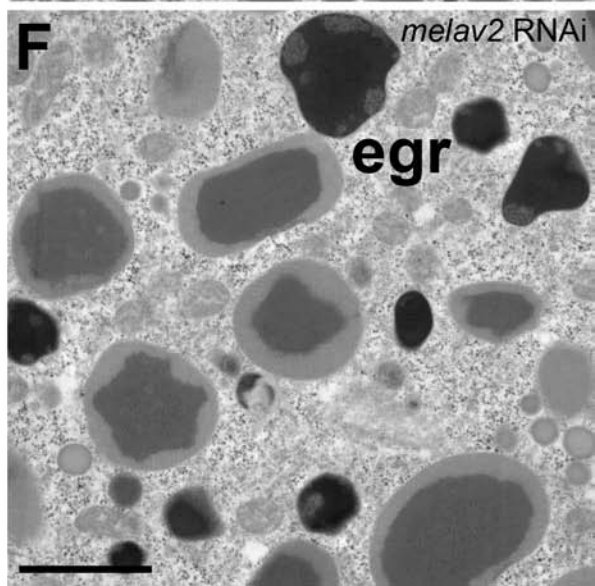

Supplement: Additional file 4 — Comparison of oogenesis of control and melav2 RNAi treated M. lignano. The oocyte of control (A) and melav2 RNAi treated (B) M. lignano exhibited comparable morphology. The ultrastructure of developing eggs (C, D) egg granules (E, F) was not affected by melav2 RNAi treatment. de, developing egg; egr, egg granules; nl, nucleolus; onu, oocyte nucleus. Scale bars: A-D 2 μm; E, F, 1 μm. [file 1471-213X-9-62-S4.PDF]
